# Severe Diabetic Ketoacidosis With Refractory Hypernatremia due to Hypokalemia‐Induced Arginine Vasopressin Resistance: A Case Report and Literature Review

**DOI:** 10.1155/carm/6883361

**Published:** 2026-06-13

**Authors:** Reem Shihab, May Shihab, Hala O. Abdallah, Suha Nori, Obay Mashaqi

**Affiliations:** ^1^ Department of Medicine, Faculty of Medicine and Health Sciences, An-Najah National University, Nablus, State of Palestine, najah.edu; ^2^ Department of Medicine, An-Najah National University Hospital, Nablus, State of Palestine, najah.edu

**Keywords:** arginine vasopressin resistance, case report, diabetic ketoacidosis, hypernatremia, hypokalemia

## Abstract

Diabetic ketoacidosis (DKA) is a life‐threatening complication of diabetes characterized by hyperglycemia, high‐anion‐gap metabolic acidosis, and ketonemia. Hypernatremia in DKA is uncommon and typically reflects disproportionate free water loss. Arginine vasopressin resistance (AVP‐R), previously known as nephrogenic diabetes insipidus, may occur in the setting of hypokalemia, leading to polyuria, hypotonic urine, and worsening hypernatremia. We report the case of a 14‐year‐old previously healthy female who presented with new‐onset severe DKA complicated by profound acidosis, hyperglycemia, hypokalemia, and hypernatremia. Despite standard DKA management, she developed persistent polyuria and rising serum sodium levels. Urine studies demonstrated dilute urine with inappropriately elevated pH, raising suspicion for hypokalemia‐induced AVP‐R. Management included aggressive potassium repletion, careful adjustment of intravenous fluids, and adjunctive desmopressin therapy. This approach resulted in normalization of electrolyte abnormalities, resolution of polyuria, and full clinical recovery. This case highlights the importance of recognizing hypokalemia‐induced AVP‐R as a reversible cause of refractory hypernatremia in DKA and underscores the need for individualized fluid and electrolyte management beyond standard treatment protocols.

## 1. Introduction

Diabetic ketoacidosis (DKA) is a life‐threatening complication of diabetes, typically occurring in Type 1 diabetes [1]. It is often triggered by the onset of a new disease, infection, or poor treatment adherence. Clinical presentation includes tachycardia, tachypnea, dehydration, Kussmaul respirations, fruity breath, and abdominal tenderness, with severe cases potentially showing altered mental status or signs of cerebral edema [1]. It is biochemically characterized by hyperglycemia (typically greater than 250 mg/dL), metabolic acidosis with a low arterial pH (< 7.3) and bicarbonate level (< 15 mEq/L), and the presence of ketonemia or ketonuria, usually accompanied by an elevated anion gap (> 14–15 mEq/L). Typical laboratory findings commonly include leukocytosis, hyponatremia, and hyperkalemia [2]. The presence of hypernatremia in DKA is considered an atypical and rare presentation, which has no clear approach to follow; the causes could potentially be explained by excessive water losses relative to the osmotic loss of sodium through the urine [3].

Arginine vasopressin resistance (AVP‐R), formerly known as nephrogenic diabetes insipidus (NDI), is characterized by impaired renal water reabsorption, leading to polyuria, dilute urine, and often hypernatremia. Laboratory findings include low urine osmolality or specific gravity despite hypernatremia and elevated plasma osmolality [4].

To our knowledge, the coexistence of severe DKA with hypokalemia, hypernatremia, and AVP‐R is rare and underreported [5, 6]. Hypokalemia‐induced NDI can exacerbate free water losses, worsening hypernatremia and dehydration, and complicating the clinical course of DKA [6]. This case report highlights this unusual presentation, emphasizes the role of hypokalemia‐induced NDI in refractory hypernatremia, and stresses the importance of careful electrolyte monitoring and fluid management in complex DKA cases.

## 2. Case Presentation

A 14‐year‐old previously healthy female presented with a 2‐day history of flu‐like symptoms, including sore throat, dry cough, fatigue, and subjective fever, followed by worsening abdominal pain and recurrent vomiting. Her family reported a 2‐month history of weight loss, decreased appetite, and recent polyuria and polydipsia. She initially presented to a referring hospital, where she was found to be tachypneic with Kussmaul breathing. Initial laboratory evaluation demonstrated severe metabolic acidosis (pH 6.9, bicarbonate ∼5 mmol/L) in the setting of marked hyperglycemia, consistent with DKA, and she was transferred to our institution for further management.

On admission, vital signs revealed blood pressure of 150/72 mmHg, heart rate of 118 bpm, respiratory rate of 25 breaths per minute, and oxygen saturation of 98% on room air. Laboratory investigations confirmed severe, high‐anion‐gap metabolic acidosis (pH 6.89, bicarbonate 2.8 mmol/L), hyperglycemia (458 mg/dL), and significant hypokalemia (2.61 mmol/L), with a serum sodium of 146 mmol/L. Urinalysis demonstrated glycosuria, ketonuria, and relatively low urine specific gravity (1.015), suggesting impaired urinary concentrating ability. Key laboratory findings during the hospital course are summarized in Table 1.

**TABLE 1:** Key laboratory findings during hospital course.

Parameter	On admission	First 24 h	At discharge	Reference range
Arterial pH	6.89	7.35	7.49	7.35–7.45
HCO_3_^-^ (mmol/L)	2.8	11.1	30.5	22–26
Random Blood Glucose (mg/dL)	458	244	—	70–140
HbA1c (%)	10.9	—	—	< 5.7 (normal), ≥ 6.5 diabetes
Sodium (mmol/L)	146	154	142	135–145
Corrected Sodium (mmol/L)	152	156	—	135–145
Potassium (mmol/L)	2.61	3.88	3.68	3.5–5.0
Chloride (mmol/L)	115	120	104	98–106
Serum Osmolality (mOsm/kg)	323	325	—	275–295
Anion Gap	Elevated	Elevated	Normal	8–12 mmol/L
Urine Ketones	+3	—	—	Negative
Urine pH	6	—	—	4.5–8.0
Urine Specific Gravity	1.015	—	—	1.005–1.030

Management was initiated with aggressive intravenous fluid resuscitation, starting with a 1000 mL bolus of Ringer’s lactate (RL) followed by maintenance infusion at 150 mL/hour. Given the presence of severe hypokalemia, potassium replacement was prioritized using intravenous potassium chloride at rates of 20–40 mEq/hour via central venous access. Insulin therapy was initially withheld and subsequently initiated once serum potassium levels reached ≥ 4.0 mmol/L. Due to profound acidemia (pH 6.89), along with delayed insulin initiation and clinical deterioration, intravenous sodium bicarbonate (200 mL) was administered as a temporizing measure. During the first 24 h, the patient developed marked polyuria (400–600 mL/hour), corresponding to an estimated urinary loss of nearly 12 L/day. In contrast, intravenous fluid administration ranged from 150 to 200 mL/hour, in addition to an initial 1000 mL RL bolus, corresponding to approximately 5.2 L of intravenous fluid intake during the first 24 h. Therefore, despite aggressive fluid resuscitation, the patient developed a substantially negative fluid balance estimated at approximately 6.8 L during the first 24 h, contributing to progressive hypernatremia, with serum sodium rising to 154 mmol/L, and reflecting the severity of the ongoing free water deficit.

In the context of severe hypokalemia, persistent polyuria, and impaired urine‐concentrating ability, a diagnosis of hypokalemia‐induced renal tubular dysfunction with partial AVP‐R was considered.

Desmopressin was initiated at a dose of 0.05 mg orally every 12 h. A temporal association was observed between correction of hypokalemia and continuation of fluid therapy with the addition of desmopressin administration and reduction in urine output (from 400 to 600 mL/hour to approximately 120–150 mL/hour), along with stabilization of serum sodium levels, supporting a functional and reversible NDI–like state.

Fluid therapy was subsequently adjusted, with a transition to dextrose‐containing fluids (5% dextrose at 150–200 mL/hour) once blood glucose decreased below 250 mg/dL, along with encouragement of oral free water intake.

Over the following 48–72 h, the patient demonstrated progressive clinical and biochemical improvement, with resolution of metabolic acidosis, normalization of electrolytes, and return to baseline mental status. She was transitioned to a subcutaneous insulin regimen and discharged in stable condition with outpatient endocrinology follow‐up.

## 3. Discussion

DKA usually presents with hyponatremia, which is often pseudohyponatremia due to hyperglycemia and ketones driving water out of cells [7]. Hypernatremia likely resulted from osmotic diuresis with disproportionate water loss relative to sodium, impaired thirst, inadequate fluid intake, and renal unresponsiveness to ADH. Previous reports of hypernatremia in DKA are uncommon and generally involve patients with pre‐existing AVP‐R, severe dehydration, or delayed presentation [6, 8]. In our case, hypernatremia was associated with AVP‐R secondary to severe hypokalemia, complicating clinical management.

A case series describes management challenges in DKA with hypernatremia, where careful fluid selection was essential to avoid further increases in serum sodium and adverse outcomes [9]. Additionally, when hypernatremia is severe, cautious correction rates are necessary to avoid osmotic demyelination or cerebral edema, as illustrated in related hypernatremia case series [9]. In our patient, hypernatremia was managed with careful administration of hypotonic solutions along with desmopressin to replace free water deficits, guided by frequent monitoring of serum sodium and osmolality. This individualized approach was necessary due to the coexistence of osmotic diuresis from hyperglycemia and impaired renal concentrating ability. Serum sodium levels normalized progressively without causing rapid shifts, and the patient’s mental status and overall fluid balance improved, highlighting the importance of individualized fluid management in complex DKA cases with concurrent electrolyte and renal concentrating abnormalities.

Co‐occurrence of congenital AVP‐R with DKA in a pediatric patient posed significant fluid management challenges due to simultaneous osmotic and ADH‐resistance diuresis [8]. Likewise, management of a patient with known NDI and DKA highlighted risks of worsening hypernatremia with conventional fluid resuscitation and the need for specialist input [6]. In contrast to these reports, our patient had no prior history of diabetes insipidus, and renal concentrating defects developed acutely. To our knowledge, this is the first description of hypokalemia‐induced AVP‐R complicating DKA.

The use of sodium bicarbonate in DKA remains controversial. In the context of our patient’s complex metabolic and electrolyte disturbances, the decision to use bicarbonate required careful consideration. Current international guidelines, including US recommendations, restrict its use to cases of severe acidemia (typically pH < 6.9), as metabolic acidosis usually improves with fluid resuscitation and insulin therapy [10]. Available evidence does not demonstrate a clear benefit in recovery time or hospital stay, while potential risks include hypokalemia, cerebral edema, paradoxical central nervous system acidosis, and reduced tissue oxygen delivery [11]. These risks are particularly relevant in patients with pre‐existing electrolyte disturbances, such as hypokalemia, as in our case. Nevertheless, severe untreated acidemia may contribute to myocardial depression, reduced vascular tone, and catecholamine resistance, supporting its selective use in life‐threatening situations [1, 12, 13]. In our patient, bicarbonate was administered as a temporizing measure in the setting of profound acidemia and delayed insulin initiation due to severe hypokalemia, with close monitoring. In parallel, fluid selection was individualized. Although 0.9% NaCl remains the traditional fluid of choice in many pediatric DKA protocols, RL was selected in this case to minimize the risk of hyperchloremic metabolic acidosis associated with large‐volume normal saline administration. Balanced crystalloids such as RL provide a more physiologic electrolyte composition with lower chloride content and lactate metabolism to bicarbonate, which may attenuate hyperchloremia and acid–base disturbances. Pediatric studies have demonstrated that RL is associated with reduced hyperchloremia and improvement in acid–base recovery, with some randomized trials suggesting a trend toward faster resolution of DKA compared with normal saline [14]. This approach was particularly important in our patient, where ongoing free water losses due to osmotic diuresis and AVP‐R required careful fluid selection and frequent adjustment.

Severe hypokalemia can induce AVP‐R by impairing the kidney’s ability to concentrate urine, primarily through autophagic degradation of aquaporin‐2 (AQP2) channels, which are essential for water reabsorption in the collecting ducts [15]. It may also contribute to renal tubular dysfunction and a hyperchloremic normal anion gap metabolic acidosis by impairing distal hydrogen ion secretion. The response to desmopressin in acquired AVP‐R is variable and likely depends on the degree of renal resistance and residual receptor function; however, partial forms may respond to supraphysiologic doses [16].

In our patient, persistent hypokalemia, inappropriately elevated urine pH despite systemic acidosis, hyperchloremia, and a nonanion gap metabolic acidosis supported the presence of renal tubular dysfunction complicating DKA. AVP‐R secondary to hypokalemia was managed with gradual correction of hypokalemia along with desmopressin administration, which contributed to the restoration of renal responsiveness with adjunctive desmopressin. Hypernatremia in DKA is uncommon and should prompt careful evaluation for AVP‐R and other causes of free‐water loss. This case highlights the importance of monitoring urine output and osmolality in patients with unexpected hypernatremia. Early recognition of hypokalemia‐induced AVP‐R is essential, as management requires tailored fluid therapy and close electrolyte monitoring beyond standard DKA protocols.

## Author Contributions

Hala O. Abdallah: writing–original draft (Introduction).

May Shihab: writing–original draft (Case Presentation).

Reem Shihab: data curation and writing–original draft (Discussion).

Suha Nori: writing–original draft (Discussion).

Obay Mashaqi: providing supervision and guidance throughout the project.

## Funding

The authors received no funding for this case report.

## Disclosure

All authors read and approved the final manuscript.

## Ethics Statement

Informed consent was obtained from the participant included in the study.

## Consent

Consent was obtained for the publication of anonymized data and any accompanying images.

## Conflicts of Interest

The authors declare no conflicts of interest.

## Data Availability

The data that support the findings of this study are available from the corresponding authors upon reasonable request.
